# Adult ovarian and sellar region mixed germ cell tumor: a case report and literature review

**DOI:** 10.3389/fonc.2024.1360158

**Published:** 2024-05-21

**Authors:** Dawei Chen, Anling Zhang, Kun Xue, Shuyu Liu, Xu Yan

**Affiliations:** ^1^ Department of Neurosurgery, The First Hospital of Jilin University, Changchun, China; ^2^ Department of Stomatology, Jilin Province First Automobile Works (FAW) General Hospital, Changchun, China; ^3^ Department of Pathology, The First Hospital of Jilin University, Changchun, China

**Keywords:** mixed germ cell tumors, ovarian tumor, sellar region tumor, alphafetoprotein, endoscopic surgery

## Abstract

Mixed germ cell tumors (mGCTs) involving both the ovaries and sellar region have been rarely reported; thus, they pose significant challenges in clinical management. Our report of a case of a 26-year-old female with left ovarian mGCTs (dysgerminoma + yolk sac tumor) who presented with postoperative headaches and blurred vision contributes new information to the literature on treating mGCTs, which can lead to standardized regimens and sequencing guidelines. A physical examination revealed right temporal hemianopia, and elevated levels of alpha-fetoprotein were detected in serum and cerebrospinal fluid. Magnetic resonance imaging (MRI) of the sellar region revealed a space-occupying lesion. Pathological examination of the tumor after endoscopic transnasal resection confirmed the diagnosis of mGCTs (germinomas + yolk sac tumor). The patient received adjuvant chemotherapy and radiotherapy at reduced dosages. During follow-up, tumor markers remained within normal limits, and there was no evidence of tumor recurrence on sellar region MRI. This case highlights the rarity of the simultaneous occurrence of ovarian and sellar region mGCTs and emphasizes the importance of accurate diagnosis and multidisciplinary management.

## Introduction

Germ cell tumors are most commonly detected in the gonads, specifically the testes and ovaries, with 1% to 5% of cases occurring outside the gonads, such as in the central nervous system, mediastinum, and sacrococcygeal region. Among them, intracranial germ cell tumors (iGCTs) are rare and heterogeneous, comprising only 1% to 3% of central nervous system tumors (1). iGCTs exhibit significant age, gender, and geographical differences, iGCTs predominantly occur along the midline, with 40%–60% arising in the pineal region and 30%–40% in the suprasellar region. Bifocal instances, where tumors appear simultaneously in both areas, account for 2%–26% of cases. iGCTs show a male predominance, with the majority (50%–70%) being germinomas located in the pineal region. Conversely, germinomas in the suprasellar region are more commonly observed in females (2, 3). Additionally, in 5%–6% of cases, iGCTs can metastasize between the brain and spinal cord (4, 5). However, the exact etiology and pathogenesis of iGCTs remain unclear.

The 2021 World Health Organization classification divided iGCTs into germinomas and non-germinomatous germ cell tumors (NGGCTs) based on clinical and pathological features. Two-thirds of all cases are germinomatous GCTs, while one-third of cases are NGGCTs. NGGCTs include embryonal carcinoma, yolk sac tumor, choriocarcinoma, teratoma (including immature, mature, and teratoma with somatic-type malignancy), as well as mixed germ cell tumors (mGCTs) (4). Mixed GCTs are composed of two or more of the subtypes, with the most common being a combination of germinoma and teratoma (6). The phenotypic characteristics of intracranial germ cell tumors, as indicated by tumor markers and immunohistochemistry, are detailed in **Table 1** (7–9). Treatment protocols and prognoses vary across different subtypes, necessitating accurate diagnosis based on the patient’s clinical symptoms, radiographic findings, serum/cerebrospinal fluid (CSF) tumor markers, and immunohistochemical features to avoid misdiagnosis and diagnostic delays.

**Table 1:** Tumor markers and immunohistochemistry of intracranial germ cell tumors.

Subtype	PLAP	AFP	hCG	CEA	OCT3/4	CD117	CD30	CK	SALL4
Germinomas	+	−	±	−	±	−	−	±	+
Teratoma	−	±	−	−	±	±	−	+	±
Yolk sac tumor	±	+	−	−	−	−	−	+	+
Embryonal carcinoma	±	±	±	+	+	−	−	+	+
Choriocarcinoma	±	−	+	−	−	−	−	+	+
Mixed germ cell tumor	Depends on components								

+, positive; −, negative; ±, positive or negative; AFP, alpha-fetoprotein; CD117, proto-oncogene c-kit; CD30, Recombinant Human CD30; CEA, carcino-embryonic antigen; CK, cytokeratin; hCG, human chorionic gonadotropin; PLAP, placental alkaline phosphatase; OCT3/4, octamer-binding transcription factor ¾; SALL4, spalt-like transcription factor 4.

Simultaneous occurrence of mGCTs in both gonadal and extragonadal sites is extremely rare. Literature reports have documented four cases of simultaneous occurrence of intracranial and testicular GCTs (10). Here, we report a case of mGCTs occurring simultaneously in the ovaries and sellar region.

## Case description

**Figure 1:**
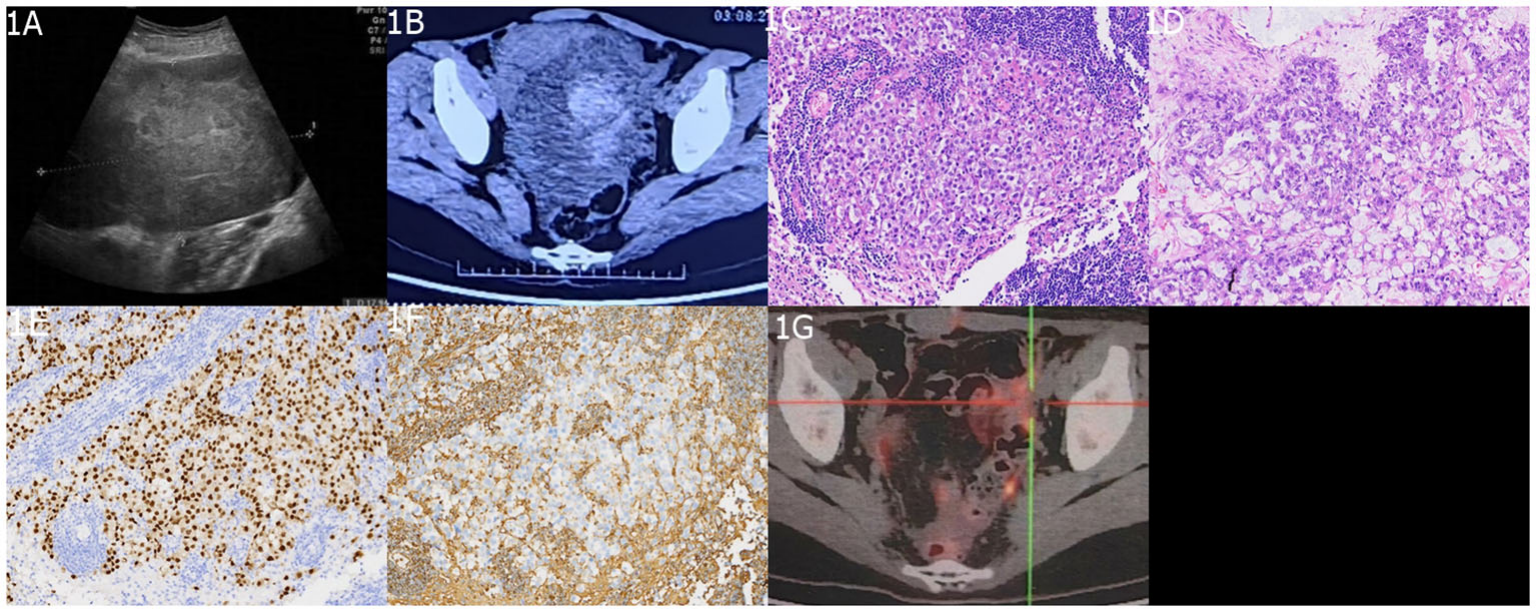
Imaging and Histopathological Findings of Ovarian mGCTs. **(A)** An ultrasound scan highlights the absence of the left ovary and reveals a 15 cm × 13 cm × 9 cm heterogeneous hypoechoic mass behind the uterus in the pelvic midline. **(B)** An abdominal enhanced CT scan depicts a large, irregularly dense mass within the abdomen and pelvis, with solid components showing slight contrast enhancement and cystic components exhibiting no enhancement. The histopathological aspects include **(C)** a slide of non-germinomatous germ cell tumor tissue and **(D)** a yolk sac tumor tissue section, both stained with hematoxylin and eosin (H&E) at ×200 magnification. Immunohistochemical staining demonstrates **(E)** CD117 positivity and **(F)** SALL4 positivity. **(G)** A postoperative 18F-FDG PET/CT scan shows postsurgical changes in the left adnexal region with no evidence of enlarged or hypermetabolic lymph nodes in the abdominal, pelvic, or retroperitoneal areas.

A 26-year-old female presented to the hospital with a one-week history of headache and blurred vision. Twenty days prior, she had sought medical attention at a local hospital for abdominal pain and a palpable mass in the abdomen. Abdominal ultrasound and CT scan revealed a pelvic mass (**Figures 1A, B**). The patient’s serum alpha-fetoprotein (AFP) level was significantly elevated at 2,000 ng/ml (normal range <20), beta-human chorionic gonadotropin (β-HCG) was 35 ng/ml (normal range 0–25), and carbohydrate antigen 125 (CA125) was 188 U/ml (normal range <35). She underwent a left ovarian tumor resection at the local hospital. Pathological examination revealed left ovarian mGCTs (80% dysgerminoma + 20% yolk sac tumor) (**Figures 1C–F**), and cytology of the ascitic fluid showed no atypical cells. Lymph node biopsies of the left fallopian tube, greater omentum, pelvic peritoneum, and para-aortic region were negative for malignancy. The patient was diagnosed with clinical stage IC2 ovarian cancer, indicating that the tumor was confined to the ovaries or fallopian tubes, with no evidence of metastasis. During an 8-day hospital stay, her serum alpha-fetoprotein (AFP) levels decreased to 810 ng/ml, and beta-human chorionic gonadotropin (β-HCG) levels reduced to 30 ng/ml. She was discharged to recuperate before commencing chemotherapy. One week prior to admission to our hospital, the patient experienced headaches and blurred vision without polydipsia or polyuria (24-hour urine volume of 1,800 ml). Cranial MRI revealed a space-occupying lesion in the sellar region.

**Figure 2:**
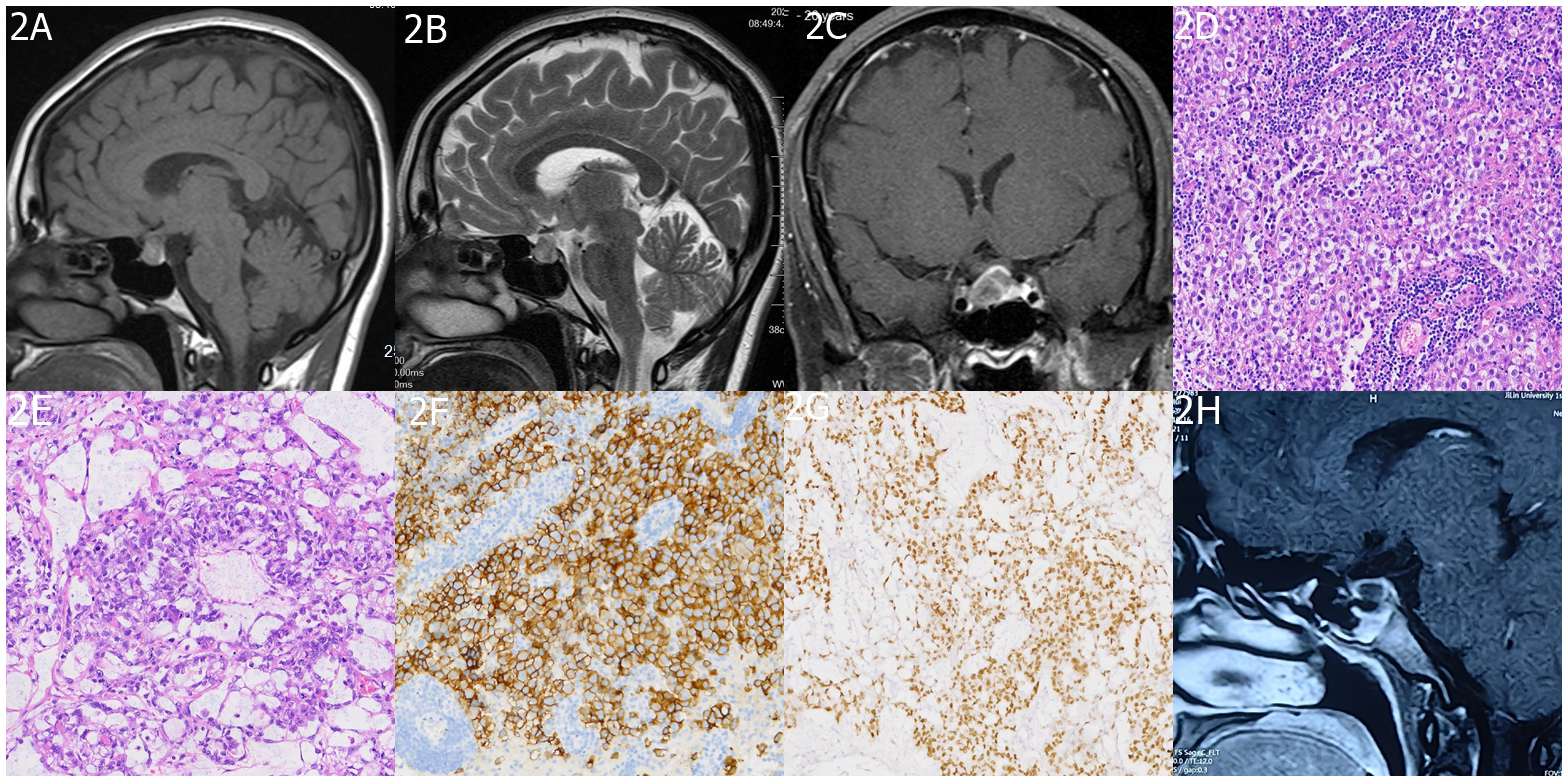
Imaging and Histopathology of Sellar mGCTs. **(A, B)** MRI images exhibit heterogeneous signal intensities in the sellar area with iso/slightly low signals on T1 and T2-weighted images. **(C)** Post-contrast scans reveal mild, uneven enhancement in the affected area. The histopathological findings are detailed in **(D)** a histological slide of the sellar germ cell tumor, and **(E)** a yolk sac tumor section, both stained with H&E at ×200 magnification. Immunohistochemical staining shows **(F)** OCT 3/4 positivity and **(G)** AFP positivity. Finally, **(H)** a follow-up MRI at 6 months post-treatment confirms no signs of tumor recurrence in the sellar region.

Physical examination showed normal visual acuity, visual fields, and bilateral fundus in the left eye, while the right eye had temporal hemianopia. The hormonal profile, thyroid function tests, and serum cortisol were within normal limits. Urinary cortisol was measured at 68 nmol/24h (normal range 108–960). Serum AFP was 400 ng/ml, β-HCG was 26 ng/ml, CA125 was 119 U/ml, CSF AFP was 510 ng/ml, and CSF β-HCG was 30 ng/ml. Cytology of the CSF showed no atypical cells. Chest computed tomography (CT) and spinal MRI were normal, while abdominal contrast-enhanced CT revealed postoperative changes in the pelvic region. Cranial MRI showed an irregular nodular lesion measuring 1.6 cm × 1.3 cm × 1.1 cm in the sellar region, with an iso- to slightly hypointense signal on T1-weighted and T2-weighted images, heterogeneous enhancement after contrast administration, compression of the optic chiasm, left deviation of the pituitary stalk (**Figures 2A–C**). The initial diagnosis upon admission was metastatic tumor in the sellar region or pituitary adenoma. Differential diagnoses included craniopharyngioma, hypothalamic glioma, Langerhans cell histiocytosis, and hypophysitis.

Following a multidisciplinary treatment consultation, the patient underwent endoscopic transsphenoidal resection of the sellar lesion. Intraoperatively, the tumor appeared grayish-brown, with evidence of old hemorrhage, firm consistency, clear boundaries from the pituitary, and abundant blood supply. The tumor was completely removed. Postoperative pathological examination confirmed mGCTs (60% germinomas + 40% yolk sac tumor) (**Figures 2D–G**). Following surgery, the patient’s headaches resolved and visual disturbances significantly improved. No surgical complications occurred. The postoperative serum AFP level was 110 ng/ml, and β-HCG, CA125, and urinary cortisol were within the normal range. CSF AFP was 130 ng/ml. PET/CT showed no abnormal metabolic activity in the sellar region or the surgical area in the left adnexa, while the rest of the body showed no abnormalities (**Figure 1G**). After the patient’s physical condition improved, she was transferred to the oncology department for chemotherapy using the PEB regimen: cisplatin 30 mg (day 1) + etoposide (VP-16) 100 mg (days 1–5) + bleomycin 15 mg (days 2 and 5), administered once a month for six cycles, and it did not include cytokine-induced killer immunotherapy. The patient underwent a full spinal MRI and CSF cytology, both of which returned normal results. During chemotherapy, prophylactic irradiation was delivered to the sellar region (30 Gy) and whole ventricles (20 Gy), while spinalirradiation was not performed. The patient underwent regular follow-up examinations and monitoring of tumor markers. After six months of follow-up, the patient’s condition remained stable; tumor markers were within normal limits; abdominal contrast-enhanced CT showed postoperative changes in the left adnexa; and sellar MRI revealed no tumor recurrence (**Figure 2H**). Long-term follow-up is ongoing.

## Discussion

mGCTs, a rare type of malignant tumor originating from the primitive germ cells within the ovaries, are composed of two or more germ cell tumor elements. These tumors are commonly diagnosed in young females and manifest as abdominal masses, abdominal pain, and amenorrhea. Elevated levels of serum AFP and β-HCG are indicative of mGCTs, and imaging studies such as abdominal ultrasound or CT may show solid or mixed cystic-solid masses. The treatment of choice is fertility-preserving tumor resection, which includes removal of the affected fallopian tube and ovary while preserving the uterus, followed by chemotherapy. The PEB regimen, consisting of bleomycin, etoposide, and cisplatin, is the chemotherapy protocol most frequently employed (11). In this particular case, the patient underwent fertility-preserving tumor resection followed by six cycles of PEB chemotherapy, and given the consideration for her physiological and reproductive well-being, radiotherapy was omitted. iGCTs are primarily found in children and adolescents, whereas in adults, they frequently signify metastatic disease originating from gonadal germ cell tumors (12). Consequently, abdominal imaging and tumor marker analysis are crucial to exclude the possibility of an ovarian or testicular primary tumor leading to brain metastasis (13). The incidence of brain metastasis in gonadal GCTs varies between 2% to 16%, as shown by diverse studies. The predominant metastatic subtypes are choriocarcinoma, yolk sac tumor, and embryonal carcinoma (14–17).

In this instance of an ovarian mGCT occurring concurrently with a sellar lesion, several factors pointed to the intracranial tumor being a primary iGCT rather than a metastatic disease. The ovarian tumor was categorized as early-stage IC2, as per FIGO staging (1), with no evidence of local or distant metastasis on imaging. The cytology was negative, and the tumor markers returned to normal postoperatively. A whole-body PET/CT scan revealed no metastatic disease, making metastasis from the ovarian primary improbable. Clinically, GCTs in the sellar region frequently mimic the presentation of pituitary adenomas, exhibiting symptoms such as diabetes insipidus, visual disturbances, and endocrine dysfunction. MRI findings typically show an iso to hypointense T1/T2 signal, heterogeneous enhancement, stalk thickening, and loss of posterior pituitary bright spot ADC values tend to be low, and spectroscopy reveals lipid peaks (18–20). AFP and β-hCG serve as useful tumor markers for iGCTs, despite ongoing debates about optimal cutoff values (21). These markers not only assist in the diagnosis but also monitor the response to treatment and potential recurrence. In this case, the markers normalized after resection, suggesting effective treatment.

The first-line treatment involves radiation therapy or chemotherapy for pure germinomas and a combination of chemotherapy and radiation for mGCTs (22). Chemotherapy drugs such as cisplatin, etoposide, and bleomycin are combined with radiation to minimize the dosage. Reduced-dose radiation therapy can effectively minimize side effects, notably endocrine disturbances and neurocognitive dysfunction. This patient underwent PEB chemotherapy and prophylactic sellar and whole ventricular radiation without craniospinal irradiation. iGCTs, encompassing various subtypes such as mGCTs, share identical histological and immunophenotypic characteristics with gonadal GCTs. Accurate pathological diagnosis plays a vital role, although it is challenging due to its similarities with pituitary adenomas. The risk of misdiagnosis increases with negative cytology and markers. In Japan, surgery/biopsy is recommended even after chemotherapy/radiation (7, 23, 24). Extensive sampling should be performed to assess all components, guiding treatment and prognosis. Treatment should be based on the most malignant component when pathology conflicts with markers. The role of surgery remains controversial. Resection is typically preferred for mature teratomas. It can also ameliorate visual deficits from sellar lesions that are unresponsive to radiation, address NGGCT components overlooked by radiation, alleviate mass effect/hydrocephalus, and manage growing teratoma syndrome. Nonetheless, previous radiation can raise surgical risks (25, 26). In this instance, aggressive resection resulted in visual improvement without complications. In this case, postoperative clinical symptoms of the patient with sellar mGCT improved swiftly, and tumor markers decreased significantly. By the 6-month follow-up, marker levels returned to normal, and sellar MRI revealed no evidence of tumor recurrence, indicating a positive treatment response. Nonetheless, ongoing long-term follow-up is essential.

In conclusion, an individualized combination of chemoradiation, based on pathology, can yield favorable outcomes in these complex tumors. A multidisciplinary approach is imperative, balancing the risks and benefits of surgery, radiation, and chemotherapy for each patient. Future studies are warranted to optimize diagnosis, treatment sequencing, and standardized protocols for mGCTs.

## Data availability statement

The raw data supporting the conclusions of this article will be made available by the authors, without undue reservation.

## Ethics statement

The studies involving humans were approved by the ethics committee of Jilin University First Hospital. The studies were conducted in accordance with the local legislation and institutional requirements. The participants provided their written informed consent to participate in this study. Written informed consent was obtained from the individual(s) for the publication of any potentially identifiable images or data included in this article.

## Author contributions

DC: Writing – review & editing. AZ: Formal analysis, Visualization, Writing – review & editing. KX: Writing – original draft. SL: Writing – original draft. XY: Formal analysis, Writing – review & editing.

## References

[1] Frappaz D Dhall G Murray MJ Goldman S Faure Conter C Allen J. Eano, Sno and Euracan consensus review on the current management and future development of intracranial germ cell tumors in adolescents and young adults. Neuro Oncol. (2022) 24:516–27. doi: 10.1093/neuonc/noab252 PMC8972311 34724065

[2] Takami H Perry A Graffeo CS Giannini C Narita Y Nakazato Y. Comparison on epidemiology, tumor location, histology, and prognosis of intracranial germ cell tumors between mayo clinic and Japanese consortium cohorts. J Neurosurg. (2020) 134:446–56. doi: 10.3171/2019.11.Jns191576 32005022

[3] Burnham EL Tomita T. Histogenesis of intracranial germ cell tumors: primordial germ cell vs. Embryonic stem cell. Childs Nerv Syst. (2023) 39:359–68. doi: 10.1007/s00381-022-05808-w 36595083

[4] Louis DN Perry A Wesseling P Brat DJ Cree IA Figarella-Branger D. The 2021 who classification of tumors of the central nervous system: A summary. Neuro Oncol. (2021) 23:1231–51. doi: 10.1093/neuonc/noab106 PMC8328013 34185076

[5] Rogers SJ Mosleh-Shirazi MA Saran FH. Radiotherapy of localised intracranial germinoma: time to sever historical ties? Lancet Oncol. (2005) 6:509–19. doi: 10.1016/s1470-2045(05)70245-x 15992700

[6] Aihara Y Watanabe S Amano K Komatsu K Chiba K Imanaka K. Placental alkaline phosphatase levels in cerebrospinal fluid can have a decisive role in the differential diagnosis of intracranial germ cell tumors. J Neurosurg. (2018) 131:687–94. doi: 10.3171/2018.3.Jns172520 30265190

[7] Takami H Perry A Graffeo CS Giannini C Daniels DJ. Novel diagnostic methods and posttreatment clinical phenotypes among intracranial germ cell tumors. Neurosurgery. (2020) 87:563–72. doi: 10.1093/neuros/nyaa108 32348488

[8] Huo Z. Germ-cell tumors of the central nervous system in Peking Union medical college hospital: A 20-year clinicopathologic review. Chin Med J (Engl). (2020) 133:240–2. doi: 10.1097/cm9.0000000000000606 PMC7028174 31868808

[9] Zhang Y Li M Liu J Deng K Zhu H Lu L. Oct3/4 is a potential immunohistochemical biomarker for diagnosis and prognosis of primary intracranial germ cell tumors: A systematic review and meta-analysis. Front Neurosci. (2023) 17:1169179. doi: 10.3389/fnins.2023.1169179 37476834 PMC10354551

[10] Han L Lu J Fang L Qi S Song Y. Simultaneous intracranial and testicular germ cell tumors: illustrative case. J Neurosurg Case lessons. (2021) 1:Case2067. doi: 10.3171/case2067 36034508 PMC9394165

[11] Armstrong DK Alvarez RD Bakkum-Gamez JN Barroilhet L Behbakht K Berchuck A. Ovarian cancer, version 2.2020, Nccn clinical practice guidelines in oncology. J Natl Compr Canc Netw. (2021) 19:191–226. doi: 10.6004/jnccn.2021.0007 33545690

[12] Singh R Fazal Z Freemantle SJ Spinella MJ. Between a rock and a hard place: an epigenetic-centric view of testicular germ cell tumors. Cancers (Basel). (2021) 13:1506–23. doi: 10.3390/cancers13071506 33805941 PMC8036638

[13] Takami H Graffeo CS Perry A Ohno M Ishida J Giannini C. Histopathology and prognosis of germ cell tumors metastatic to brain: cohort study. J Neurooncol. (2021) 154:121–30. doi: 10.1007/s11060-021-03810-x 34272633

[14] Le A Arbab M Adra N Miller JC Watson GA Shiue K. Literature review of management of brain metastases from germ cell tumors. Chin Clin Oncol. (2022) 11:14. doi: 10.21037/cco-21-127 35400165

[15] McAlpine K Clark R Jiang M Hansen A Hemminki O Hamilton RJ. Case - intra-abdominal metastases following ventriculoperitoneal shunt insertion for primary intracranial germ cell tumor. Can Urological Assoc J = J l’Association Des urologues du Canada. (2022) 16:E496–e8. doi: 10.5489/cuaj.7895 PMC9484745 35426790

[16] Lo AC Hodgson D Dang J Tyldesley S Bouffet E Bartels U. Intracranial germ cell tumors in adolescents and young adults: A 40-year multi-institutional review of outcomes. Int J Radiat Oncol Biol Phys. (2020) 106:269–78. doi: 10.1016/j.ijrobp.2019.10.020 31654785

[17] Maekawa K Tokumitsu T Minematsu E Noguchi H Nakamura E Asada Y. Cervical lymph node metastasis of ovarian dysgerminoma: A case report with fine needle aspiration cytology. Diagn Cytopathol. (2020) 48:356–9. doi: 10.1002/dc.24351 31886634

[18] Takami H Graffeo CS Perry A Giannini C Daniels DJ. Epidemiology, natural history, and optimal management of neurohypophyseal germ cell tumors. J Neurosurg. (2020) 134:437–45. doi: 10.3171/2019.10.Jns191136 32032947

[19] Yang M Wang J Zhang L Liu J. Update on mri in pediatric intracranial germ cell tumors-the clinical and radiological features. Front Pediatr. (2023) 11:1141397. doi: 10.3389/fped.2023.1141397 37215600 PMC10192609

[20] Li Y Wang P Zhang J Li J Chen L Qiu X. Multiparametric framework magnetic resonance imaging assessment of subtypes of intracranial germ cell tumors using susceptibility weighted imaging, diffusion-weighted imaging, and dynamic susceptibility-contrast perfusion-weighted imaging combined with conventional magnetic resonance imaging. J Magn Reson Imaging. (2022) 56:1232–42. doi: 10.1002/jmri.28132 35278008

[21] Takami H Graffeo CS Perry A Giannini C Nakazato Y Saito N. Impact of tumor markers on diagnosis, treatment and prognosis in cns germ cell tumors: correlations with clinical practice and histopathology. Brain Tumor Pathol. (2023) 40:124–32. doi: 10.1007/s10014-023-00460-x PMC10113344 36995447

[22] Denyer S Bhimani AD Patil SN Mudreac A Behbahani M Mehta AI. Treatment and survival of primary intracranial germ cell tumors: A population-based study using seer database. J Cancer Res Clin Oncol. (2020) 146:671–85. doi: 10.1007/s00432-019-03088-7 PMC11804720 31745701

[23] Sahay A Epari S Chinnaswamy G Chatterjee A Goda JS Patil V. Primary intracranial germ cell tumors: A study with an integrated clinicopathological approach. Neurol India. (2023) 71:500–8. doi: 10.4103/0028-3886.378644 37322747

[24] Takami H Graffeo CS Perry A Giannini C Nakazato Y Saito N. Roles of tumor markers in central nervous system germ cell tumors revisited with histopathology-proven cases in a large international cohort. Cancers (Basel). (2022) 14:979–89. doi: 10.3390/cancers14040979 35205726 PMC8869781

[25] Yeo KK Nagabushan S Dhall G Abdelbaki MS. Primary central nervous system germ cell tumors in children and young adults: A review of controversies in diagnostic and treatment approach. Neoplasia. (2023) 36:100860. doi: 10.1016/j.neo.2022.100860 36521378 PMC9772847

[26] Nakamura H Takami H Yanagisawa T Kumabe T Fujimaki T Arakawa Y. The Japan society for neuro-oncology guideline on the diagnosis and treatment of central nervous system germ cell tumors. Neuro Oncol. (2022) 24:503–15. doi: 10.1093/neuonc/noab242 PMC8972225 34671804

